# Sperm Cholesterol Content Modifies Sperm Function and TRPV1-Mediated Sperm Migration

**DOI:** 10.3390/ijms22063126

**Published:** 2021-03-18

**Authors:** Luca De Toni, Iva Sabovic, Vincenzo De Filippis, Laura Acquasaliente, Daniele Peterle, Diego Guidolin, Stefania Sut, Andrea Di Nisio, Carlo Foresta, Andrea Garolla

**Affiliations:** 1 Unit of Andrology and Reproduction Medicine, Department of Medicine, University of Padova, 35128 Padova, Italy; luca.detoni@unipd.it (L.D.T.); stefania.sut@studenti.unipd.it (S.S.); andrea.dinisio@unipd.it (A.D.N.); carlo.foresta@unipd.it (C.F.); 2 Department of Clinical and Experimental Sciences, University of Brescia, 25123 Brescia, Italy; iva.sabovic@gmail.com; 3 Department of Pharmaceutical and Pharmacological Sciences, University of Padova, 35131 Padova, Italy; vincenzo.defilippis@unipd.it (V.D.F.); laura.acquasaliente@unipd.it (L.A.); danifire90@gmail.com (D.P.); 4 Department of Neurosciences, Institute of Human Anatomy, University of Padova, 35121 Padova, Italy; diego.guidolin@unipd.it

**Keywords:** thermotaxis, sperm membrane, cholesterol recognition amino acid consensus sequence, molecular modeling, epicholesterol

## Abstract

Transient receptor potential channels-vanilloid receptor 1 (TRPV1) regulates thermotaxis in sperm-oriented motility. We investigated the role of membrane cholesterol (Chol) on TRPV1-mediated human sperm migration. Semen samples were obtained from five normozoospemic healthy volunteers. Sperm membrane Chol content, quantified by liquid chromatography-mass spectrometry, was modified by incubating cells with 2-hydroxypropyl-ß-cyclodextrin (CD) or the complex between CD and Chol (CD:Chol). The effect on sperm migration on a 10 μM capsaicin gradient (CPS), a TRPV1 agonist, was then investigated. Motility parameters were evaluated by Sperm Class Analyser. Intracellular calcium concentration and acrosome reaction were measured by staining with calcium orange and FITC-conjugated anti-CD46 antibody, respectively. TRPV1-Chol interaction was modelled by computational molecular-modelling (MM). CD and CD:Chol, respectively, reduced and increased membrane Chol content in a dose-dependent manner, resulting in a dose-dependent increase and reduction of sperm migration in a CPS gradient. MM confirmed a specific interaction of Chol with a TRPV1 domain that appeared precluded to the Chol epimer epicholesterol (Epi-Chol). Accordingly, CD:Epi-Chol was significantly less efficient than CD:Chol, in reducing sperm migration under CPS gradient. Chol inhibits TRPV1-mediated sperm function by directly interacting with a consensus sequence of the receptor.

## 1. Introduction

The gain of sperm-oriented motility of sperm cells within the female reproductive tract is a key event contributing to the fertility potential [1]. At least three main mechanisms have been recognized to drive sperm cells through the female reproductive tract. The first is the oriented swimming of the sperm cell against the genital fluid flow, known as rheotaxis [2]. Chemotaxis also drives the directional changes of the sperm movement towards the source of a chemoattractant source, such as progesterone, according to its concentration gradient [3,4]. However, the most studied long-range drive for spermatozoa is thermotaxis, allowing the cell to move according to a temperature gradient from the cooler site of sperm deposition to the warmer site of possible oocyte fertilization [5]. So far, a redundancy of thermoreceptors has been acknowledged in the regulation of thermotaxis, but recent findings highlighted a major role of transient receptor potential channels (TRP) and, in particular, the vanilloid receptor 1 member of TRP family named TRPV1 [6,7]. TRPV1 is recognized as a temperature-sensitive nonselective ion channel, since the increase in temperature progressively enhances the chances of the receptor activation [8]. In addition, the temperature threshold of activation can be lowered by a number of nonthermal agonists, such as the low pH, ethanol, lidocaine and, most importantly, the pepper vanilloid capsaicin (CPS) [9].

Another factor recognized to influence sperm motility is the fine composition of the sperm plasma membrane and, in particular, the membrane’s cholesterol (Chol) content. It is widely acknowledged that, upon spermiation and during the epididymal transit, the sperm surface undergoes a consistent reduction in sperm cholesterol content according to the gain of sperm fertilizing ability [10,11,12,13,14]. In addition, once ejaculated into the female genital tract, sperm membranes are further depleted of Chol because of the high local content of bicarbonate and albumin [15]. These membrane rearrangements have been associated with the acquisition of cell forward motility and capacitation, a key step for acrosome reaction and subsequent fusion with the oocyte [16,17,18]. Importantly, membrane Chol is recognized to be unevenly distributed into structural and kinetic domains or pools hosting several membrane proteins. The cholesterol content of these domains is thought to actively affect the function of the membrane proteins located therein [19,20]. Despite the fact that this model is also applied to explain the variation of sperm activity according to the cell Chol content, the molecular mechanisms of these processes are still under-investigated. Interestingly, recent studies identified a specific domain, named the cholesterol recognition amino acid consensus (CRAC) sequence, within TRPV1 [21]. CRAC sequences represent selective binding site for the Chol involved in the modulation of protein activity. Accordingly, the binding of Chol to the CRAC sequence of TRPV1 was found to reduce CPS-activated current in somatic cell models transfected with TRPV1 gene [21].

The aim of this study was to clarify the role of Chol on TRPV1-mediated sperm migration. To this end, we experimentally modified the membrane Chol content of human spermatozoa, by the use of a cyclodextrin-based delivery approach, and evaluated the effect on sperm function and migration towards a CPS gradient. In addition, we investigated the possible involvement of the CRAC motifs in the observed effect of the Chol.

## 2. Results

### 2.1. Efficacy of Cyclodextrin in Depleting and Increasing the Cholesterol Content of Sperm Membrane

**Figure 1:**
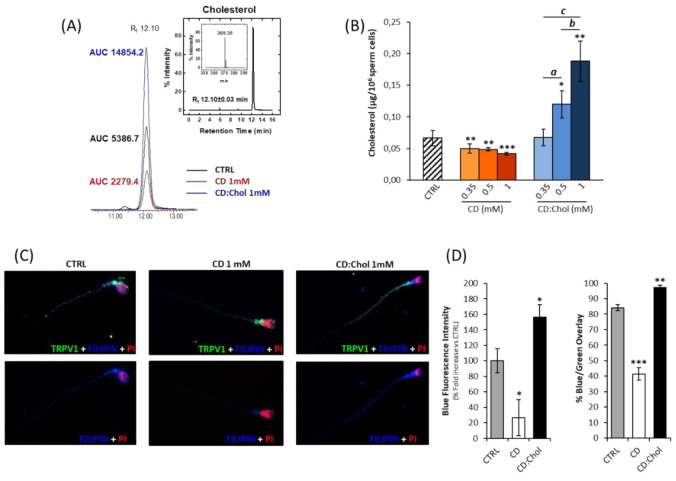
Experimental modification of the membrane cholesterol content with cyclodextrin approach. (**A**) Representative images of ultrahigh performance liquid chromatography-mass spectrometry (UPLC-MS) analysis of cholesterol (Chol) content in naïve sperm cells (CTRL), in sperm cells incubated with 2-hydroxypropyl-ß-cyclodextrin (CD) or with the complex between CD and Chol (CD:Chol), both at the concentration of 1 mM. The chromatographic peaks are flanked by the respective values of area under the curve. The inset shows a representative chromatogram of Chol standard and the corresponding high-resolution MS spectrum with a monoisotopic mass value of 369.35 atomic mass units (a.m.u). (**B**) UPLC-MS quantification of Chol in sperm cells incubated with CD or CD:Chol, at concentrations ranging from 0 (CTRL) to 1 mM. Data are expressed as µg of Chol per 10^6^ sperm cells. Significance: * = *p* < 0.05, ** = *p* < 0.01, *** = *p* < 0.001 vs. CTRL; a = *p* < 0.05, b = *p* < 0.01 and c = *p* < 0.001 between the indicated conditions. (**C**). Immunofluorescence staining of TRPV1 (green) and cholesterol, detected by Filipin II (blue), in naïve sperm cells (CTRL) and cells treated with 1 mM CD or 1 mM CD:Chol. Cell nuclei were counterstained with propidium iodide (PI, red). The upper panels show the merging of the three colors’ channels, whilst in the lower panel the green channel was omitted for clarity. In (**D**) the image analysis is reported. The left plot shows the blue fluorescence staining intensity, reported as %-fold increase compared to CTRL; the right plot shows the percentage of overlay between the green (TRPV1) and the blue channels (Chol). Significance: * = *p* < 0.05, ** = *p* < 0.01, *** = *p* < 0.001 vs. CTRL.

In order to modify the Chol content in the sperm membrane, 2-hydroxypropyl-ß-cyclodextrin was used as a molecular carrier of hydrophobic species as previously described [22]. Accordingly, the efficacy of this approach was investigated (Figure 1). Sperm cells samples form from healthy normozoospermic donors were incubated for 30 min with either CD or CD:Chol, at a concentration ranging from 0 (CTRL) to 1 mM. The quantification of total Chol content was performed by ultrahigh performance liquid chromatography-mass spectrometry (UPLC-MS) and whose representative chromatograms are reported in Figure 1A. With the increase in CD concentration in the culture medium, a significant decrease in total sperm Chol content was detected (respectively: 0.066 ± 0.003 µg/10^6^ cells CTRL vs. 0.050 ± 0.007 µg/10^6^ cells CD 0.35 mM; 0.049 ± 0.002 µg/10^6^ cells CD 0.5 mM and 0.042 ± 0.002 µg/10^6^ CD 1 mM; all *p* values < 0.01. Figure 1B). On the other hand, incubation with CD:Chol at a concentration equal to or greater than 0.5 mM was associated with a significant increase in total Chol content (respectively, 0.120 ± 0.022 µg/10^6^ cells CD:Chol 0.5 mM, *p* = 0.013 vs. CTRL, and 0.188 ± 0.032 µg/10^6^ cells CD:Chol 1 mM, *p* < 0.001 vs. CTRL. Figure 1B). In order to assess the cell site of Chol depletion or increase associated with the incubation with, respectively, CD or CD:Chol, a double-staining immunofluorescence approach was performed. In particular, Chol was visualized by the use of Filipin III dye, a polyene macrolide fluorescent antibiotic that selectively binds to Chol [23].

The proportion of Chol localized on sperm membrane was estimated through image analysis on the base of the overlap between the Filipin III staining (blue) with the TRPV1 signal (green), visualized by immunofluorescence, which is recognized as a membrane protein [7] (Figure 1C). Importantly, the treatment with either CD or CD:Chol was not associated with any significant variation of TRPV1 staining compared to the untreated control (all *p* values > 0.05, data not shown). In agreement with the results obtained by UPLC-MS, incubation with 1 mM CD was associated with a significant reduction in Filipin III staining intensity compared to untreated control (100.0 ± 15.6% CTRL vs. 26.7 ± 23.4% CD; *p* = 0.011), whilst incubation with 1 mM CD:Chol resulted in a significant increase in Filipin III signal (156.2 ± 16.1% CD:Chol, *p* = 0.012; Figure 1D). Importantly, the variations in Filipin III fluorescence intensity were found to be essentially related to the membrane, as evidenced by the, respectively, reduced and increased overlap with the TRPV1 signal (84.1 ± 1.9% CTRL; 41.3 ± 4.0% CD, *p* = 0.0004 vs. CTRL; 97.1 ± 1.7% CD:Chol, *p* = 0.001 vs. CTRL).

Taken together, these results indicate that CD is an efficient method for modifying the Chol content of the sperm membrane at will.

### 2.2. Effect of Membrane Chol Content on TRPV1-Mediated Sperm Migration and Function

**Figure 2:**
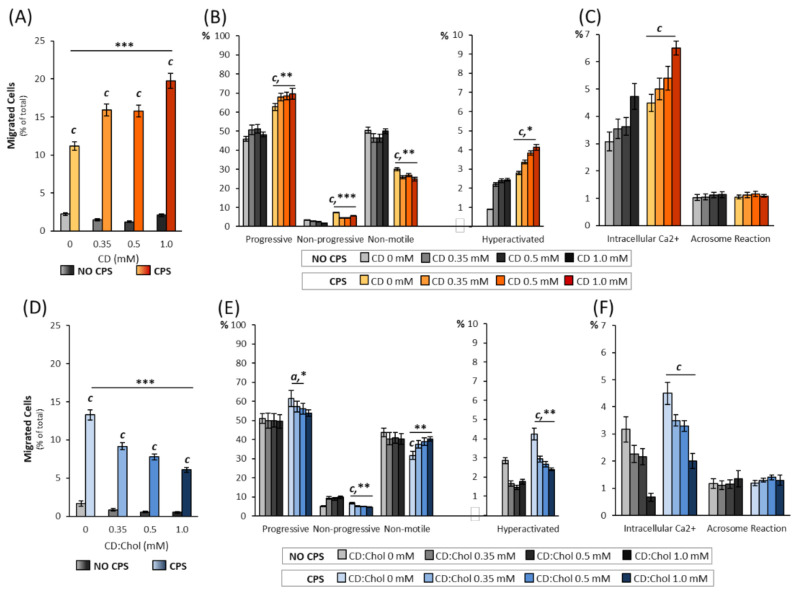
Effect of membrane Chol content on TRPV1-mediated sperm migration and function. (**A**). Relative quantification of sperm cells migrated in absence of gradient (NO CPS) or towards a 10 μM gradient of capsaicin (CPS), previously incubated with 2-hydroxypropyl-ß-cyclodextrin (CD) at concentrations ranging from 0 (CTRL) to 1 mM, as detailed in the methods section. Data are reported as percentages of migrated cells. Significance: c = *p* < 0.001 vs. the corresponding NO CPS condition; *** = *p* < 0.001 for the cumulative effect of CD + CPS vs. CD + NO CPS. (**B**). Motility categories of sperm cells incubated at different concentrations of CD and migrated towards NO CPS or CPS gradient. Samples were evaluated by automated Sperm Class Analyser (SCA) and are reported as percentages. Significance: c = *p* < 0.001 vs. the corresponding NO CPS condition; * = *p* < 0.05, ** = *p* < 0.01 and *** = *p* < 0.001 for the cumulative effect of CD + CPS vs. CD + NO CPS. (**C**). Evaluation of sperm intracellular calcium (Intracellular Ca^2+^) levels and acrosome reaction, obtained by fluorescence microscopy and by flow cytometry, respectively, in sperm cells incubated at different concentrations of CD and migrated towards NO CPS or CPS gradient. Data are reported as percentages of, respectively, calcium orange positive cells and CD46-positive cells. Significance: c = *p* < 0.001 vs. the corresponding NO CPS condition. (**D**). Relative quantification of sperm cells migrated in NO CPS or CPS gradient, previously incubated with the complex between CD and Chol (CD:Chol) at concentrations ranging from 0 (CTRL) to 1 mM, as detailed in the methods section. Data are reported as percentages of migrated cells. Significance: c = *p* < 0.001 vs. the corresponding NO CPS condition; *** = *p* < 0.001 for the cumulative effect of CD + CPS vs. CD + NO CPS. (**E**) Motility categories of sperm cells incubated at different concentrations of CD:Chol and migrated towards NO CPS or CPS gradient. Samples were evaluated by automated Sperm Class Analyser (SCA) and are reported as percentages. Significance: a = *p* < 0.05; c = *p* < 0.001 vs. the corresponding NO CPS condition; * = *p* < 0.05 and ** = *p* < 0.01 for the cumulative effect of CD + CPS vs. CD + NO CPS. (**F**) Evaluation of sperm intracellular Ca^2+^ levels and acrosome reaction in sperm cells incubated at different concentrations of CD:Chol and migrated towards NO CPS or CPS gradient. Data are reported as percentages of, respectively, calcium orange positive cells and CD46-positive cells. Significance: c = *p* < 0.001 vs. the corresponding NO CPS condition.

In order to evaluate the effect of membrane Chol content on TRPV1-mediated sperm migration and function, sperm cell samples form healthy normozoospermic donors were incubated for 30 min with either CD or CD:Chol, at concentrations ranging from 0 mM (CTRL) to 1 mM, and then submitted to an accumulation test under a gradient of 10 μM capsaicin (CPS). The random cell drift was also evaluated with the accumulation test in the absence of a CPS gradient (NO CPS, Figure 2A). In the NO CPS condition, sperm migration was generally below the 3% and incubation with CD, regardless on its concentration, had no significant effect on the percentage of the accumulated cells. On the other hand, in the CPS gradient condition, a strong and significant increase in the migration rate was observed, whose extent was further increased by the incubation with CD in a concentration-dependent manner (respectively, 11.18 ± 0.56% CTRL; 15.91 ± 0.80% CD 0.35 mM; 15.78 ± 0.79% CD 0.5 mM, 19.77 ± 0.99% 1 mM; all *p* values < 0.001 vs. corresponding NO CPS; *p* < 0.001 for the cumulative effect CD + CPS vs. CD + NO CPS Figure 2A). The motility parameters of the migrated sperm cells were also assessed (Figure 2B). Compared to the cells migrated in NO CPS conditions, the cells migrated towards a CPS gradient had significantly higher percentages of progressive motility, nonprogressive motility and hyperactivated cells, but lower levels of immotile sperms (all *p* < 0.001 vs. NO CPS CTRL). Incubation with CD was associated with further significant increase in the percentages of cells with nonprogressive (*p* < 0.001 for cumulative effect CD + CPS vs. CD + NO CPS) and hyperactivated motility (*p* < 0.001 for cumulative effect CD + CPS vs. CD + NO CPS). An opposite trend was observed for nonmotile cells (*p* = 0.016 for cumulative effect CD + CPS vs. CD + NO CPS; Figure 2B). The detailed analysis of the motility parameters obtained by the Sperm Class Analyser revealed that, compared to the NO CPS condition, cells migrated towards the CPS gradient had significantly higher values of VLC, VSL, VAP, ALH, and WOB (*p* < 0.05 for WOB; *p* < 0.001 for the remaining parameters vs. NO CPS CTRL). The combination of incubation with CD and migration towards the CPS gradient resulted in further dose-dependent increases for VSL, VAP, ALH and LIN (*p* < 0.05 for LIN and *p* < 0.01 for the remaining parameters the cumulative effect of CD + CPS vs. CD + NO CPS, Supplemental Figure S1A). In addition, the migration towards the CPS gradient was generally associated with increased intracellular calcium content compared to the corresponding migration without CPS (*p* < 0.001 vs. NO CPS CTRL; Figure 2C). Incubation with CD was associated with a concentration-dependent increase in intracellular calcium, that, however, was not amplified by the cumulative interaction with the CPS gradient. Differently, the extent of acrosome reaction was largely unaffected by the both the migration towards CPS gradient and/or the incubation with CD regardless of its concentration (*p* = 0.002 vs. NO CPS), being generally very low, with percentages of reacted cells below around 1% (Figure 2C).

The effect of the increased Chol content on the sperm membrane was then evaluated by the use of the CD:Chol complex as a delivery tool. The results of the accumulation assay are reported in Figure 2D. The low rate of drift observed in the NO CPS condition showed a trend towards a further lowering after the incubation with CD:Chol (data not shown). As expected, when cells were allowed to migrate towards the CPS gradient, the percentage of accumulated cells was significantly increased (*p* < 0.001 vs. NO CPS CTRL). However, incubation with CD:Chol was associated with a progressive and significant blunting of the migration rate, whose extent was strictly correlated with the concentration of CD:Chol (respectively, 13.30 ± 0.86% CTRL; 9.15 ± 0.46% CD:Chol 0.35 mM; 7.80 ± 0.39% CD:Chol 0.5 mM; 6.10 ± 0.31% CD:Chol 1 mM; *p* < 0.001 for the cumulative effect of CD:Chol + CPS vs. CD:Chol + NO CPS Figure 2D). In particular, the analysis of the motility parameters in cells accumulated towards the CPS gradient showed that the percentages of nonprogressive and hyperactivated cells were significantly reduced by incubation in CD:Chol, in a concentration-dependent manner (respectively: *p* < 0.001 and *p* = 0.004 for the cumulative effect of CD:Chol + CPS vs. CD:Chol + NO CPS).

Coherently, the nonmotile sperm population showed a significantly inversed trend (*p* = 0.002 for cumulative effect CD:Chol + CPS vs. CD:Chol + NO CPS; Figure 2E). The evaluation of the detailed sperm motility parameters, assessed by Sperm Class Analyzer, showed that the cells migrated towards the CPS gradient had a significant reduction in the VLC and ALH parameters (respectively: *p* = 0.011 and *p* < 0.001 for the cumulative effect CD:Chol + CPS vs. CD:Chol + NO CPS; Supplementary Figure S1B). Finally, the incubation with CD:Chol was associated with a progressive reduction of the intracellular calcium but no significant cumulative effect of the CPS gradient on the CD:Chol concentration was observed (*p* = 0.932 for cumulative effect CD:Chol + CPS vs. CD:Chol + NO CPS; Figure 2F). Once again, the rate of the acrosome reaction was mainly unaffected by the migration towards the CPS gradient after the incubation with CD:Chol (Figure 2F).

Taken together, these results suggest that the membrane Chol content acts as a fine tuner of the TRPV1-mediated sperm migration.

### 2.3. Computational Modelling of the Interaction between Cholesterol and TRPV1

**Figure 3:**
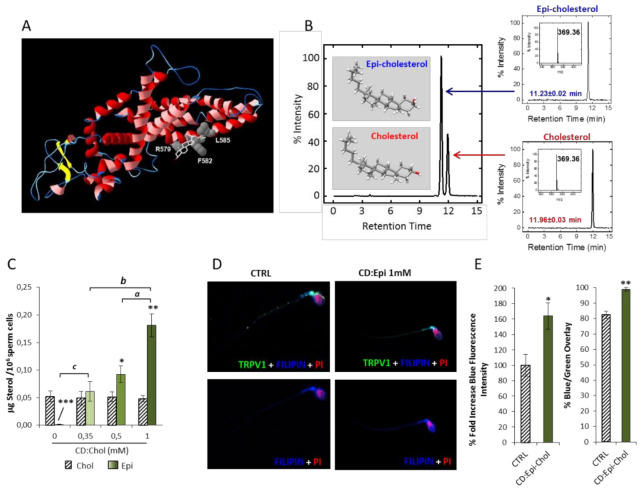
Computational modelling of TRPV1–cholesterol interaction and experimental addition of epicholesterol onto sperm membrane with cyclodextrin approach. (**A**) Representative image of the interaction between TRPV1 (red ribbon structure) and cholesterol (white sticks structure) at computational modeling. Major amino acids expected to be involved in molecular binding are reported as balls side chains and marked with their initials and position. (**B**) Representative images of ultrahigh performance liquid chromatography-mass spectrometry (UPLC-MS) analysis of cholesterol (Chol) and epicholesterol (Epi-Chol) content in naïve sperm cells (CTRL) incubated with complex between 2-hydroxypropyl-ß-cyclodextrin and Epi-Chol (CD:Epi-Chol) at the concentration of 1 mM. The chromatographic peaks are flanked by representative chromatogram of Chol and Epi-Chol standards and the corresponding high-resolution MS spectra, both showing a monoisotopic mass value of 369.35 a.m.u. (**C**) UPLC-MS quantification of Chol and Epi-Chol in sperm cells incubated with CD:Epi-Chol, at concentrations ranging from 0 (CTRL) to 1 mM. Data are expressed as µg of Chol or Epi-Chol (sterols) per 10^6^ sperm cells. Significance: * = *p* < 0.05, ** = *p* < 0.01, *** = *p* < 0.001 vs. the corresponding Chol bar; a = *p* < 0.05, b = *p* < 0.01 and c = *p* < 0.001 between the indicated conditions. (**D**). Immunofluorescence staining of TRPV1 (green) and sterols, detected by Filipin II (blue), in naïve sperm cells (CTRL) and cells treated with 1 mM CD:Epi-Chol. Cell nuclei were counterstained with propidium iodide (PI, red). The upper panels show the merging of the three colors channels whilst, in the lower panel, the green channel was omitted for clarity. In (**E**) the image analysis is reported. The left plot shows the blue fluorescence staining intensity, reported as %-fold increase compared to CTRL; the right plot shows the percentage of overlay between the green (TRPV1) and the blue channels (Chol). Significance: * = *p* < 0.05 and ** = *p* < 0.01 vs. CTRL.

Cholesterol is acknowledged to influence the function of membrane proteins through two main mechanisms [24]. The first, less-specific mechanism relies on the rigid tetracyclic structure of Chol which, by limiting the conformational degrees of freedom of the neighboring phospholipids, reduces the fluidity of cell membrane and, in turn, forces integral membrane proteins to change conformation [25]. The second, more specific mechanism relies on the properties of the defined structural motifs of membrane proteins, able to bind Chol with variable affinity to the hydroxyl group, to the multirings system, to the aliphatic tail by van der Waals forces and/or a combination of the three [26]. Particularly for TRPV1, a Chol consensus motif has been identified in a cleft of the protein structure predicted to be at the interface region with cell membrane [21]. By the application of a computational analysis, we confirmed that the most energetically favorable predicted docking site for Chol in TRPV1 was found in the S5 helix domain and involved Arg579, Phe582 and Leu585 residues, with a ligand efficiency index of 0.30 (Figure 3A), where the ligand efficiency index, defined as the ΔG score (kcal/mol)/nonhydrogen atoms, represents an estimate of the ligand-receptor binding efficiency. Importantly Epi-Chol, the α-3-OH-cholesterol epimeric form, was expected to display an energetically less favorable interaction with the TRPV1 binding domain, compared to Chol, because of the increased van der Waals volume of the α-face due to the different position of the OH group. Accordingly, differently from Chol, Epi-Chol demonstrated ineffectiveness in modifying the capsaicin evoked current in HEK293 cells transfected with the *Trpv1* gene, suggesting a major inhibitory effect of Chol based on the stereospecific interaction with TRPV1 binding site [21]. On this basis, we investigated the possible persistence of a similar molecular pattern for Chol on TRPV1-mediated sperm function.

**Figure 4:**
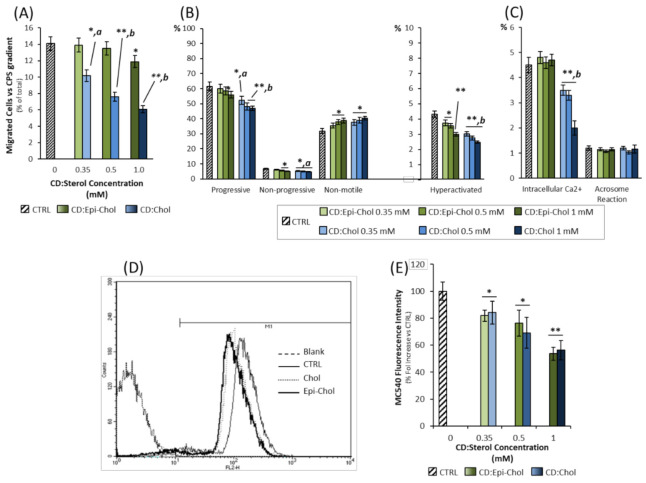
Differential effect of epicholesterol and cholesterol on TRPV1-mediated sperm migration and function. (**A**). Relative quantification of sperm cells migrated in a 10 μM gradient of capsaicin, previously incubated with either the complex between 2-hydroxypropyl-ß-cyclodextrin and epicholesterol (CD:Epi-Chol) or the complex between 2-hydroxypropyl-ß-cyclodextrin and epicholesterol (CD:Chol) (blue), at concentrations ranging from 0 mM (CTRL) to 1 mM. Data are reported as percentages of migrated cells. Significance: * = *p* < 0.05 and ** = *p* < 0.01 vs. CTRL; a = *p* < 0.05 and b = *p* < 0.01 vs. the corresponding CD:Epi-Chol condition. (**B**). Motility categories of sperm cells previously incubated at different concentrations of CD: Epi-chol or CD:Chol, and then migrated towards the CPS gradient. Data were obtained by automated Sperm Class Analyser (SCA) and are reported as percentages. Significance: * = *p* < 0.05 and ** = *p* < 0.01 vs. CTRL; a = *p* < 0.05 and b = *p* < 0.01 vs. the corresponding CD:Epi-Chol condition (**C**). Evaluation of sperm intracellular calcium (Intracellular Ca^2+^) levels and acrosome reaction, obtained by fluorescence microscopy and by flow cytometry, respectively, in sperm cells incubated at different concentrations of CD:Epi-Chol or CD:Chol and then migrated towards the CPS gradient. Data are reported as percentages of, respectively, calcium orange positive cells and CD46-positive cells. Significance: ** = *p* < 0.01 vs. CTRL; b = *p* < 0.01 vs. the corresponding CD:Epi-Chol condition. (**D**). Representative staining of Merocyanine 540 (MC540), evaluated by flow cytometry, of naïve sperm cells (CTRL) or cells incubated with CD:Epi-Chol or CD:Chol 1 mM. In blank condition, MC540 was omitted. In (**E**) the plot shows the fluorescence staining intensity for MC540 in sperm cells incubated with either CD:Epi-Chol or CD:Chol at concentration ranging from 0 (CTRL) to 1 mM. Data are reported as % fold increase compared to CTRL. Significance: * = *p* < 0.05 and ** = *p* < 0.01 vs. CTRL.

As for Chol, 2-hydroxypropyl-ß-cyclodextrin was used as a molecular carrier to add Epi-Chol to the sperm membrane and the efficacy of this process was firstly investigated. Sperm cells samples from healthy normozoospermic donors were incubated for 30 min with the complex CD:Epi-Chol, at a concentration ranging from 0 (CTRL) to 1 mM. The total content of Chol and Epi-Chol was quantified by UPLC-MS, whose representative chromatograms and mass spectra are reported in Figure 1B. As expected, Epi was nearly undetectable in naïve cells from CTRL (0.0011 ± 0.0005 μg/10^6^ cells) but the incubation with CD:Epi-Chol at increasing concentration was associated with a progressive and significant increase in the total Epi content (respectively: 0.061 ± 0.018µg/10^6^ cells CD:Epi-Chol 0.35 mM, *p* = 0.0070 vs. CTRL; 0.092 ± 0.015 µg/10^6^ cells CD:Epi-Chol 0.5 mM, *p* < 0.001 vs. CTRL; 0.181 ± 0.021 CD:Epi-Chol 1 mM *p* < 0.001 vs. CTRL. Figure 3C). In order to address the cell localization of Epi-Chol delivered by the complex CD:Epi-Chol, the aforementioned double-staining immunofluorescence approach with TRPV1 was performed. The proportion of Epi localized on sperm membrane was estimated by image analysis on the base of the overlap between the Filipin III staining with the TRPV1 signal (Figure 4C). In agreement with the results obtained by UPLC-MS, incubation with 1 mM CD:Epi-Chol was associated with a significant increase in Filipin III staining intensity compared to the untreated control (100.0 ± 14.3 CTRL vs. 164.2 ± 17.1% CD:Epi-Chol; *p* = 0.0076). In particular, the variations in Filipin III fluorescence intensity were found to be essentially related to the membrane, as evidenced by the increased overlap with the TRPV1 signal (82.4 ± 2.3% CTRL; 41.3 ± 4.0% CD:Epi-Chol, *p* = 0.0004 Figure 3D,E).

### 2.4. Differential Effect of Epicholesterol and Cholesterol on TRPV1-Mediated Sperm Migration and Function

The differential effect of incubation with CD:Epi-Chol or CD:Chol on TRPV1-mediated sperm migration in a CPS gradient was then assessed (Figure 4A). In general, incubation with either CD:Epi-Chol or CD:Chol was associated with a reduction in the migratory activity.

However, for CD:Epi-Chol, a significant reduction in migrated cells compared to CTRL was observed only at the highest concentration of 1 mM (14.1 ± 0.8% CTRL vs. 11.9 ± 0.7% CD:Epi-Chol 1 mM; *p* = 0.026). Differently to CD:Chol, the reduced rate of migration was significant from the lowest concentration of 0.35 mM (respectively: 10.2 ± 0.6% CD:Chol 0.35 *p* = 0.0028; 7.6 ± 0.45% CD:Chol 0.5 mM *p* < 0.001; 6.08 ± 0.36% CD:Chol 1 mM, *p* < 0.001 all vs. CTRL; Figure 4A). Accordingly, the analysis of the sperm motility categories in cells migrated towards the CPS gradient showed that incubation with both CD:Epi-Chol and CD:Chol induced a slight but significant dose-dependent decrease in the percentages of nonprogressive cells in comparison to CTRL cells. Differently, incubation with CD:Chol was associated with a significant and dose-dependent reduction of progressive, nonprogressive and hyperactivated cells (Figure 4B). In terms of the detailed sperm motility parameters, measured by Sperm Class Analyzer, incubation with CD:Epi-Chol was associated with reduced values of VLC only at the highest concentration of 1 mM. Differently, all parameters, with the exception of WOB, were negatively and dose-dependent influenced by the corresponding CD:Chol treatment (Supplemental Figure S1C). The evaluation of the functional status of the sperm cells showed that intracellular calcium was significantly reduced by incubation with CD:Chol in a concentration-dependent manner (*p* < 0.001 vs. CTRL), whereas CD:Epi-Chol did not significantly alter this parameter. Furthermore, the levels of acrosome reaction showed no significant variation, regardless of the incubation with either CD:Epi-Chol or CD:Chol (Figure 4C).

In order to evaluate whether the effect observed for Epi and Chol on sperm migration relied on a differential influence in sperm biophysical properties, the sperm membrane fluidity was analyzed by the fluorescent probe MC540 (Figure 4D). As expected, the delivery of either Epi or Chol was associated with a reduction in membrane fluidity, as depicted by the reduction in MC450 staining intensity. However, no obvious difference was detectable for the two sterols delivered at matched concentration (Figure 4E).

Taken together, these results suggest that the influence of membrane Chol on TRPV1-mediated sperm migration is the likely result of the major specific interaction of the sterol with a corresponding TRPV1-binding domain, rather than a nonspecific influence on the overall membrane’s biophysical properties.

## 3. Discussion

In this study we provide evidence that membrane cholesterol acts as an inhibitory factor on TRPV1-mediated sperm migration, previously recognized as a major factor involved in sperm thermotaxis. In addition, through a combined computational and experimental approach, we found that the inhibition of TRPV1 does not rely on the mere reduction of cell membrane fluidity but is likely associated with a specific interaction with a Chol recognition amino acid consensus sequence of the receptor.

The importance of Chol for sperm function has been acknowledged since the late 1970s and is supported by the wide variety of homeostatic mechanisms involved in its trafficking and fine regulation of membrane content, accompanying sperm maturational changes from early spermatogenesis to the fertilization process [27,28]. However, the exact molecular targets of cholesterol action on the sperm membrane have been not completely elucidated. Until recently, the available studies mainly relied on the nonspecific modulation of the membrane’s physical properties operated by Chol [29,30]. In particular, the acquisition of motility, associated with a decreased Chol content and a more fluid plasma membrane, has been linked to easily affordable deformations during flagellar bending [13]. Beside this general effect, the Chol content was also found to modulate membrane protein function by shifting the energetic cost of protein conformational changes by the alteration of intrinsic bilayer physical properties, such as thickness, curvature, and elastic moduli [31]. These changes would influence the intracellular signal transduction and signaling pathways ultimately controlling sperm motility and activation [32,33]. In the last decade, many efforts have been devoted to the study of the lipids’ effects on the function of ion channels. In this regard, lipids are no longer thought to exert effects only on membrane elasticity properties and signaling pathways but rather to directly interact with ion channels via a fine tuning of their activity [34,35,36,37]. However, the direct interactions of Chol with sperm membrane proteins have been poorly investigated and no specific Chol target has been identified so far.

In this study we provide evidence that membrane Chol content influences TRPV1-mediated sperm functions. Moreover, by the differential effect exerted by the two stereo-isomers cholesterol and epicholesterol, we show that TRPV1 is likely a direct target of the Chol action. Importantly, in order to avoid any possible confounding effect of concurrent signaling pathways on sperm thermotaxis, we adopted a 10 µM capsaicin gradient as specific agonist of TRPV1, with this concentration being recognized as an optimal compromise between channel stimulation and apoptotic triggering [7]. In addition, we chose cyclodextrins as a molecular carrier to remove and load Chol into the sperm membrane compartment because of their high affinity for hydrophobic species and compatibility with cell models compared to other Chol acceptors such as lipoproteins and albumin [38,39]. It should be noted that increased intracellular Ca^2+^ content and hyperactivation are signs of early capacitation events strictly associated with Chol redistribution and loss from the sperm head [39]. However, we could not observe major variation of acrosome reaction levels due to the increase or depletion of Chol content by cyclodextrin treatment. From a mechanistic point of view, it should be noted that acrosome reaction in mammals is strictly dependent on the upstream activation of the cAMP/PKA system [40]. On the other hand, the cAMP/PKA system has recently been involved in the upstream phosphorylation of TRPV1, which is associated with the sensitization of the receptor to activation stimuli [41]. Our results are consistent with this sequential order of cell events and allow us to tentatively exclude a major role of sperm capacitation on the effect exerted by Chol loss on TRPV1-mediated function. Coherently, we showed that Chol loading reduced sperm migration proportionally to the concentration of the Chol–cyclodextrin complex. Despite the fact that the treatment was effective both for sperm migrated toward the CPS gradient and in the absence of gradient, the sperm migrated toward CPS were more largely affected by Chol addition, suggesting that a high Chol content in the sperm membrane likely inhibits CPS-induced TRPV1 activation. This evidence highly correlates with recent data from our group showing that infertile patients with impaired sperm parameters also displayed increased levels of membrane Chol [23].

Our results suggest a possible implication also in the field of urological oncology. In fact, TRPV1 has been recognized as part of the molecular repertoire of prostate cancer (PC) [42]. Intriguingly, Venier et al. showed that CPS was able to both reduce PC cells’ proliferation and to increase their sensitivity to radiotherapy through the suppression of the NFkB signaling [43]. Thus, as the stimulation of TRPV1 exerts cytostatic effects on PC, its inhibition is expected to have the opposite effect. This hypothesis correlates well with the available clinical evidence showing a significant association between metabolic dyslipidemia and PC progression, likely having the common root of the inhibition of TRPV1 by the increased levels of blood cholesterol [44,45].

The present study gains particular relevance once transposed to a physiological context. In fact, according to the proposed model, sperm enriched in membrane Chol are expected to have lower chances of progressing in the oviduct and successfully reaching the fertilization site although their migration is oriented and stimulated by efficient guidance mechanisms. In addition, the CRAC sequence of TRPV1 is an attractive structural moiety for the development of a possible pharmacological strategy to improve the sperm oriented motility.

## 4. Materials and Methods

### 4.1. Chemicals

Cholesterol, Epicholesterol (Epi-Chol), 2-hydroxypropyl-ß-cyclodextrin (CD), Superclean TM LC-Si SPE Tube 1 mL (Supleco), Filipin III from Streptomyces filipinensis, propidium iodide (PI) and Merocyanine 540 (MC540) were all purchased from Sigma Aldrich (Merck Group, Milan, Italy). Sperm washing medium (SWM) was purchased by Irvine Scientific (Santa Ana, CA, USA). Capsaicin was purchased from Millipore (Merck). Calcium OrangeTM AM was purchased from Thermo Fisher Scientific (Milan, Italy). Mouse monoclonal FITC-conjugated antihuman CD46 antibody was purchased from BD-Biosciences (Milan, Italy). Rabbit polyclonal anti-TRPV1 antibody was purchased from Alomone Labs (Jerusalem, Israel). FITC-conjugated antirabbit IgG, produced in goat, was purchased from Santa Cruz Biotechnology. All other reagents, solvents and salts were purchased from Fluka AG or Merck (Darmstadt, Germany).

### 4.2. Semen Samples

The study was conducted in accordance with the principles of the Declaration of Helsinki and has been formally approved by the local Hospital Ethics Committee with protocol 2208P and subsequent amendments. Each participant signed an informed consent form before participation in the study, which was performed at the Unit of Andrology and Reproductive Medicine, University of Padova. Semen samples was obtained from 5 normozoospermic healthy volunteers (mean age 35.3 ± 4.5 years), and examined according to the WHO criteria [46]. Exclusion criteria were the absence of previous genital tract infections, cryptorchidism, testicular torsion or varicocele. Donors underwent semen collection by masturbation into sterile containers after 2–5 days of sexual abstinence. Samples were allowed to liquefy for 30 min. All samples had normal viscosity and appearance, and the volume was recorded. Sperm cells concentration was measured by Makler^®^ counting chamber (Santa Ana, CA, USA) with a phase contrast microscope (Nikon Eclipse 50i, Nikor, Firenze, Italy) at 20× magnification, and the total sperm count was calculated (concentration per total volume of ejaculate).

The WHO parameters of sperm motility were evaluated using a computerized sperm analysis software, the Sperm Class Analyser (SCA, Microptic S.L., Barcelona, Spain) as detailed below. Furthermore, sperm samples underwent processing in order to obtain membrane cholesterol depletion or enrichment. After the treatment, sperm cells suspensions were divided into aliquots and used, respectively, for functional assays and sterol extraction and quantification by UPLC-MS (Waters Xevo-G2S Q-TOF, mass spectrometer, Milford, MA, USA).

### 4.3. Analysis of Sperm Motility and Kinetic Parameters

The motility parameters were carried out by the computerized motion analysis system Sperm Class Analyser. An aliquot of 10 µL from each sample was placed onto a Makler counting chamber and analyzed by a microscope connected to the computer housing the SCA software. Briefly, at every scan, five or six randomly selected microscopic fields were captured, corresponding approximately to 200 spermatozoa as suggested by WHO criteria. The performed measurements comprised (1) the WHO sperm motility categories, expressed as percent of progressively motile (PR: spermatozoa moving actively, either linearly or in a large circle, regardless of speed), nonprogressive motile (NP: sperm with all other patterns of motility with an absence of progression, e.g., swimming in small circles) and nonmotile (NM: featured by no movement) sperm cells; (2) mean kinematic parameters of motile cells: curvilinear velocity (VLC, the time-averaged velocity of the sperm head along its actual trajectory), straight-line velocity (VSL, the time-averaged velocity of the sperm head along a straight line from its first position to its last position), average path velocity (VAP), amplitude of lateral head displacement (ALH), linearity of a curvilinear path (LIN), straightness (STR), wobble (WOB), beat-cross frequency (BCF). In addition, also hyperactivated motility (HYPER) was estimated by the SCA system as the fraction of sperm cells featured by increased path velocity, decreased linearity, increased amplitude of lateral head displacement and flagellar whiplash movement compared to forward motility, characteristic of capacitated cells. For the evaluation of spermatozoa hyperactivation, the SCA cut-off points were: capture 50 frames per second, curvilinear velocity (VCL) >150 μm/s, linearity (LIN) <40% and amplitude of lateral head displacement (ALH) >3.5, as suggested elsewhere [47].

### 4.4. Semen Processing and Membrane Cholesterol-Content Modification by Cyclodextrins

After preliminary semen evaluation, the sample volume was divided into 4 equal aliquots, 3 of which intended for treatments and 1 for controls. All semen aliquots were washed once with SWM by centrifugation at 2500 rpm for 10 min to separate seminal plasma from the cells. In Chol depletion experiments, sperm cell pellets were incubated with CD solutions in SWM at the concentration of 0.35 mM, 0.5 mM or 1 mM, respectively. In Chol addition experiments, Chol was delivered to sperm membrane as a water soluble complex with CD (CD:Chol), prepared as previously described by Purdy and Graham [48] with slight modifications. Cells were then incubated with CD:Chol solutions in SWM at the concentration of 0.35 mM, 0.5 mM or 1 mM, respectively. In control samples, cells were resuspended in 1 mL of SWM, with the addition of no other reagent. All sperm cell suspensions, at an almost consistent number of 50.0 ± 3.8 × 10^6^ cells, were incubated with CD or CD:Chol for 30 min at room temperature and then washed with SWM to remove the excess. Cells destined for accumulation assays were diluted in 500µL of fresh SWM, whereas samples for cholesterol quantification were washed twice in phosphate buffered saline (PBS), centrifuged and pellets stored at −80 °C until use. The same procedure was applied to add sperm cells membrane with Epi-Chol, the 3α-OH-stereoisomer of cholesterol, previously included in a complex with CD (CD:Epi-Chol).

### 4.5. Evaluation of Sperm Migration by Accumulation Assay

The evaluation of sperm migration towards a CPS gradient was performed by the accumulation assay as previously described [7]. The assay uses a device appositely engineered for thermoseparation [49]. Briefly, it consists of a tube (38 mm total length) internally subdivided into three compartments, two chambers at the extremities, one for loading and one for recovery (respectively, the starting and the end point of sperm migration), and a compartment for the translocation in-between. For detecting sperm responses to the CPS gradient, 500 µL of each sperm sample prepared as described above was placed into the loading chamber. A CPS solution in SWM at the final concentration of 10 µM was then poured into the recovery chamber. The intermediate compartment was then filled with SWM. Capsaicin was allowed to diffuse towards the loading chamber for a standard time of 5 min. After a capsaicin gradient was established, sperm cells were allowed to migrate for 30 min at room temperature, at the end of which, sperm retrieved (or accumulated) in the recovery chamber were counted and evaluated for motility parameters as previously described. Negative controls, in which CPS was omitted, were used to evaluate the natural drift of spermatozoa in absence of a CPS gradient (NO CPS). Accumulation assay and assessment of sperm parameters were performed in triplicate, involving a different donor for each of the three separate repetitions. Donors were requested to provide two distinct sessions of donation, at few days of distance from each other, in order to perform the experiments in CPS and NO CPS gradient conditions.

### 4.6. Lipid Extraction and Liquid Chromatography-Mass Spectrometry Analysis

The total lipid fraction was extracted from the pellet of sperm cells with chloroform/methanol mixture as previously described [50]. Lyophilized lipid extracts were resuspended in 200 μL of chloroform. The sterol fraction was isolated by solid-phase extraction (SPE) on a 1-mL silica column, eluted with acetone and lyophilized in a Vacufuge^®^-Concentrator plus. The lyophilized sterol fraction was dissolved in methanol and 20 μL of this solution were analyzed by reverse-phase (RP) liquid-chromatography coupled to high-resolution mass spectrometry (LC-MS). A Poroshell 120 EC-C18 column (4.6 mm × 150 mm, 2.7-μm particle size) from Phenomenex (Torrance, CA, USA) was equilibrated and eluted at 40 °C with an acetonitrile/methanol solution (65:35 *v/v*), at a flow rate 0.3 mL/min using an Agilent (Santa Clara, CA, USA) 1290 Infinity Ultrahigh Performance Liquid Chromatography (UPLC) system, equipped with a 920 autosampler and connected to a Waters (Milford, MA, USA) Xevo-G2S Q-TOF mass spectrometer. Ionization and vaporization of the effluent from the column was obtained by atmospheric pressure ionization (APCI) in the positive ion mode. For selected applications, the mass spectrometer was operated by selecting the m/z value for each analyte and recording the total ion current (TIC) at the detector within a mass window of ± 1 atomic mass units (a.m.u). The capillary potential was set at 1.50 kV and source temperature at 110 °C. Chol and Epi-Chol in sperm samples were identified by comparing the retention time and mass spectrum (i.e., m/z value and isotopic pattern) with authentic external standards of Chol and their quantification of these analytes in extracts from sperm samples was performed using the corresponding calibration curves, obtained by analyzing increasing amount of standard Chol, under identical analytical and instrumental conditions. LC-MS data were acquired and integrated using the Mass-Lynx 4.1 software (Waters). Monoisotopic mass values were determined at a resolution >35,000 and an accuracy <2 ppm.

### 4.7. Docking

Molecular structures of Chol were retrieved from Pubchem database [51]. The experimentally assessed structure of the TRPV1 receptor was obtained from PDB database (PDB code: 5IRZ).

Receptor and ligand models were then prepared for docking by using the “Dock prep” module available in the UCSF Chimera package [52]. This procedure involved the addition of hydrogens, charges and bond order assignment. The prepared molecules and protein were then docked using the docking protocol provided by the Swissdock service (Basel, Switzerland) [53].

### 4.8. Evaluation of Intracellular Calcium, Acrosomal Reaction and Membrane Fluidity

The percentage of sperm cells with high intracellular calcium levels was estimated by staining with the long-wavelength calcium indicator Calcium Orange™, a fluorescent dye that increases the fluorescence emission intensity upon binding to calcium [54]. Briefly, sperm cells were washed twice in PBS and then incubated with Calcium Orange at the final concentration of 10 μM in PBS, for 30 min at room temperature in the dark. After washing in PBS, cells were finally smeared onto slides and a minimum of 200 spermatozoa were observed at epifluorescence microscope at 1000× magnification (Nikon Eclipse 50i; Nikon Instruments, Florence, Italy). Results were reported as the percentage of sperms positive to Calcium Orange.

Cells undergoing acrosome reaction were assessed by flow cytometry using the FITC-labelled mouse antihuman CD46 antibody as previously described [55]. Spermatozoa were first incubated for 30 min at room temperature with 100 µL PBS: BSA (Bovine Serum Albumin) to block nonspecific sites, followed by incubation with labelled anti-CD46 antibody (1:50 in PBS) for 30 min incubation at room temperature in the dark. Cells were finally incubated with PI the final concentration of 25 µg/mL in order to discriminate viable from dead cells. Samples were analyzed on a FACS Scan flow cytometer (DB Biosciences, Milan, Italy). Spermatozoa undergoing acrosome reaction were reported as the percentage of PI negative/CD46 positive cells.

Plasma membrane fluidity was assessed with MC540 probe [56]. Briefly, DMSO-stock solution of MC540 was diluted in sperm suspension at the final concentration of 4 μM and incubated for 15 min at 37 °C in the dark. Cells were finally analyzed by BD FACS Scan as previously described [57].

### 4.9. Immunofluorescence and Filipin III Staining

Sperm cells suspension were retrieved from migration experiments and fixed in 4% paraformaldehyde (PFA) in PBS for 30 min at room temperature. Cells were then smeared on superfrost plus microscope glass slides (Menzel-Gläser, Braunschweig, Germany). Samples were saturated with 5% bovine serum albumin (BSA)/5% normal donkey serum (NDS) in PBS for 30 min at room temperature. Afterwards, sperm were incubated with anti-TRPV1 antibody (1:50 in PBS) overnight at 4 °C. In negative controls, primary antibodies were omitted. Primary immunoreaction was detected by incubation with FITC-conjugated antirabbit IgG for 1 h at room temperature. In order to visualize cholesterol/localization in sperm cells, cells were stained with Filipin III, which is a polyene macrolid fluorescent antibiotic selectively binding to cholesterol [58]. In detail, cells were incubated for 2 h with Filipin III (stock solution 5 mg/mL in DMSO) at final dilution of 1:100. Slides were finally counterstained with propidium iodide (PI), mounted with antifade buffer and visualized with Video-Confocal Microscope (VICO, Nikon Instruments, Florence, Italy).

### 4.10. Statistical Analysis

Statistical analysis of the data was conducted with SPSS 21.0 for Windows (SPSS, Chicago, IL, USA). Data are expressed as means ± standard deviation (SD) and report the mean values of three independent experiments. The Kolmogorov–Smirnov test was used to check for normality of distribution. Variables not showing normal distribution were log transformed. Multivariate analysis of variance was performed to test for differences in measured parameters between groups. Main effects included treatment (free or cholesterol-loaded cyclodextrin), CPS stimulus and their interaction; post hoc analysis was performed to test differences between presence and absence of CPS at each tested cyclodextrin concentration with Bonferroni correction for multiple comparisons. *p* values <0.05 were considered as statistically significant.

## 5. Conclusions

In conclusion, our data confirmed the role of Chol as a key regulator in sperm motility and suggest TRPV1 as a molecular target of Chol action in the modulation of sperm function. Our results may also find possible applications in the diagnosis and treatment of infertile patients. In particular, the combination of cyclodextrin treatment with the exposure to CPS or temperature gradients may represent a useful tool to improve the selection of viable and functional spermatozoa. This is particularly the case of oligoasthenozoospermic patients, previously characterized by the high membrane Chol content, that frequently require assisted reproduction techniques [23]. It should be noted that, in these patients, the presence of sperm DNA fragmentation is a common feature and represents a major limitation for the success of in vitro fertilization [59]. Further studies will be addressed to ascertain the nuclear integrity in sperm cells selected through a temperature or a TRPV1-agonist gradient.

## Data Availability

Not applicable.

## Supplementary Materials

Supplementary Figure S1 is available online at https://www.mdpi.com/1422-0067/22/6/3126/s1. Figure S1: Motility parameters of sperm cells assessed by SCA. Abbreviations: curvilinear velocity (VLC), straight-line (rectilinear) velocity (VSL), average path velocity (VAP), amplitude of lateral head displacement (ALH), linearity (LIN), straightness (STR as VSL/VAP), wobble (WOB as VAP/VLC), beat-cross frequency (BCF). (A) Parameters of sperm cells migrated in absence of gradient (NO CPS) or towards a 10 μM gradient of capsaicin (CPS), previously incubated with 2-hydroxypropyl-ß-cyclodextrin (CD) at concentrations ranging from 0 (CTRL) to 1 mM, as detailed in the methods section. Significance: a = *p* < 0.05 and b = *p* < 0.01 vs. the corresponding NO CPS condition; * = *p* < 0.05 and ** = *p* < 0.01 for the cumulative effect of CD + CPS vs. CD + NO CPS. (B) Parameters of sperm cells migrated in NO CPS or CPS conditions, previously incubated with the complex between CD and cholesterol (Chol) at concentrations ranging from 0 (CTRL) to 1 mM, as detailed in the methods section. Significance: a = *p* < 0.05 vs. the corresponding NO CPS condition; * = *p* < 0.05 for the cumulative effect of CD + CPS vs. CD + NO CPS. (C) Parameters of sperm cells migrated in CPS conditions and previously incubated with CD:Chol or the complex between CD and epicholesterol (Epi-Chol) at concentrations ranging from 0 (CTRL) to 1 mM, as detailed in the methods section. Significance: * = *p* < 0.05 vs. CTRL; a = *p* < 0.05 vs. the corresponding CD:Epi-chol condition.
