# Supplementary figures and images for: Sperm Cholesterol Content Modifies Sperm Function and TRPV1-Mediated Sperm Migration
Source: Int J Mol Sci. 2021 Mar 18;22(6):3126. doi: 10.3390/ijms22063126 (PMC8003190; doi:10.3390/ijms22063126)

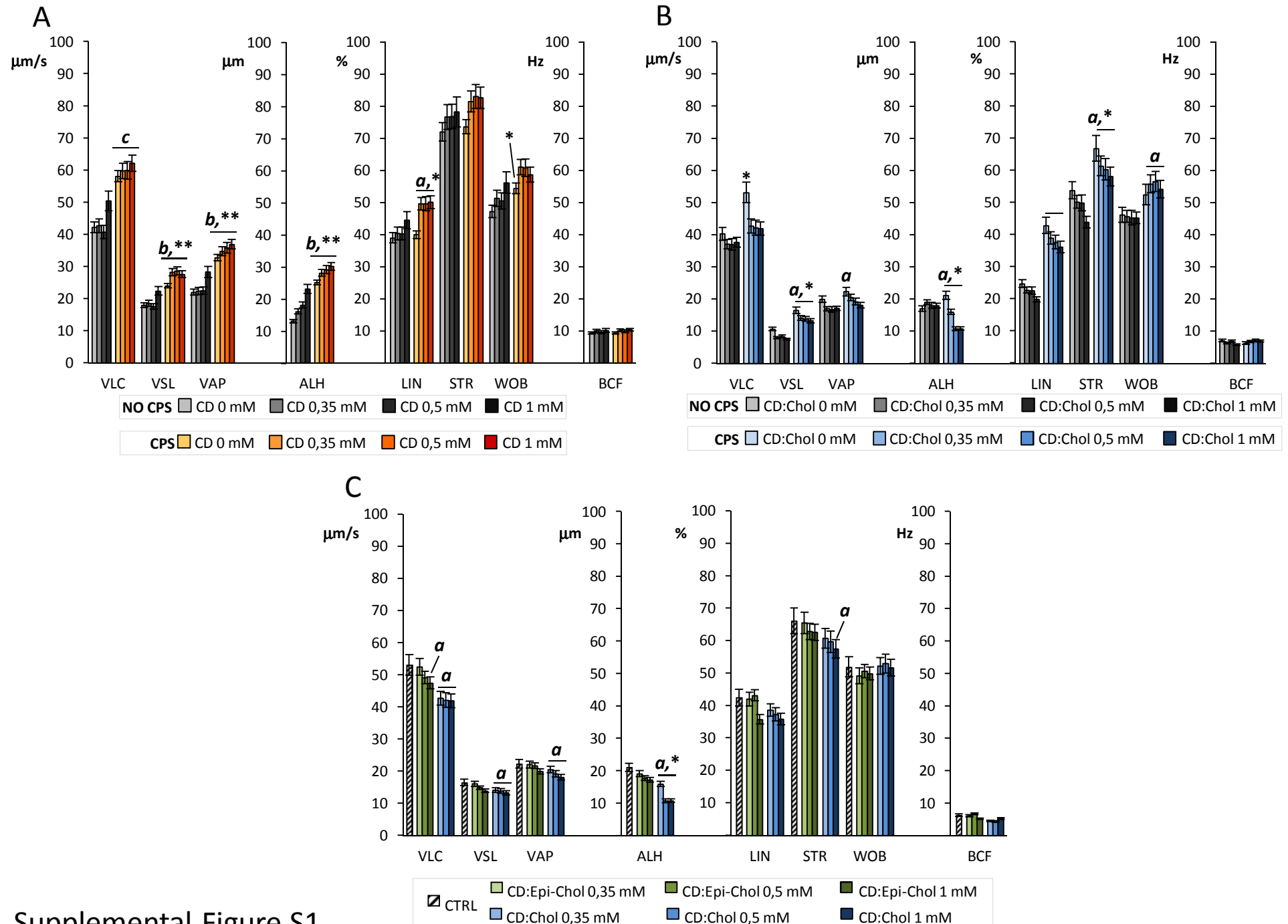

Supplement: Supplementary file 1 [file ijms-22-03126-s001.pdf]
